# Antimicrobial Functionalization of Silicone-graft-poly(*N*-vinylimidazole) Catheters

**DOI:** 10.3390/molecules29102225

**Published:** 2024-05-09

**Authors:** Luis Enrique Navarrete-Germán, Belén Gómez-Lázaro, Felipe López-Saucedo, Emilio Bucio

**Affiliations:** Departament of Radiation Chemistry and Radiochemistry, Institute of Nuclear Sciences, National Autonomous University of Mexico, Circuito Exterior s/n, Ciudad Universitaria, Mexico City C.P. 04510, Mexico; luis.german1209@gmail.com (L.E.N.-G.); belenlazaro98@gmail.com (B.G.-L.)

**Keywords:** ampicillin, device, gamma radiation, medical grade, polymers, quaternization, silver

## Abstract

In this work, we present the modification of a medical-grade silicone catheter with the *N*-vinylimidazole monomer using the grafting-from method at room temperature and induced by gamma rays. The catheters were modified by varying the monomer concentration (20–100 vol%) and the irradiation dose (20–100 kGy). Unlike the pristine material, the grafted poly(*N*-vinylimidazole) chains provided the catheter with hydrophilicity and pH response. This change allowed for the functionalization of the catheters to endow it with antimicrobial features. Thus, the quaternization of amines with iodomethane and bromoethane was performed, as well as the immobilization of silver and ampicillin. The inhibitory capacity of these materials, functionalized with antimicrobial agents, was challenged against *Escherichia coli* and *Staphylococcus aureus* strains, showing variable results, where loaded ampicillin was amply better at eliminating bacteria.

## 1. Introduction

Catheters, hydrogel patches, gauze, or sutures are materials for medical and healthcare use that aim to help the patient’s recovery. Creating new materials to improve their performance for medical use is not an easy task, as several safety standards, international regulations, and biocompatibility features must be fulfilled [1,2]. There are several methods to improve medical supplies and sanitary materials, such as surface coating, copolymerization, chemical functionalization, and derivatizations, which have proven to enhance chemical activity by adding specific functional groups to confer properties of interest [3,4], such as anticorrosive protection [5], antifouling [6], or stimulus-responsive capacity [7].

Currently, biocontamination of medical devices that are in direct contact with the patient is not uncommon in hospitals, so the incorporation of antimicrobial agents in said devices is a practical way to prevent septicemia [8,9,10] and counteract its unwanted effects. Bacteria, such as *Escherichia coli* (Gram-negative) and *Staphylococcus aureus* (Gram-positive), are pathogens associated with various gastrointestinal and urinary tract infections (*E. coli*) [11,12] and lung or bloodstream infections (*S. aureus*) [13,14]. Fortunately, a timely antibiotic dose [15,16], such as ampicillin [17], Ag(0) particles [18,19], or quaternary imidazolium salts [20,21], can eliminate these pathogens or limit their population before they cause serious health problems.

**Figure 1:**
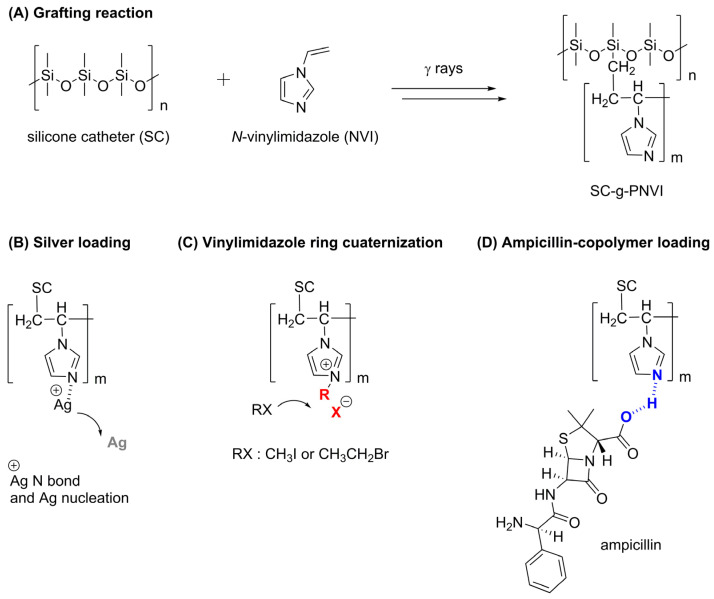
(**A**) Reaction scheme of the silicone catheter graft poly(*N*-vinylimizadole) copolymer (SC-g-PNVI) in a reaction promoted by gamma rays. (**B**) Loading Ag particles through Ag(I) photoinduced nucleation. (**C**) Quaternization of the imidazole ring with alkyl halides iodomethane and bromoethane. (**D**) Ampicillin loading via noncovalent interactions.

Medical-grade silicone is a synthetic polymer used for the manufacturing of disposables as well as biomedical devices, such as implants, inhalers, masks, and catheters, because it is highly resistant and biocompatible [22]. Silicone or polydimethylsiloxane is a linear polymer composed of Si, O, and two methyl groups (Figure 1A). The chains of the polymer have different sizes; while low-molecular-weight silicones of short chains form liquids or semi-solid materials, high-molecular-weight silicones of longer chains form materials in a solid state. So, higher-molecular-weight silicone is suitable for catheter manufacturing.

Pristine silicone may serve as the matrix for a catheter, but its surface is unable to provide antimicrobial features; adding another material on the surface can help to obtain new materials with the desired characteristics. Grafting copolymerization is a compatible method for modifying silicone surfaces. Gamma-radiation-induced grafting is a preferable method over chemical or thermally induced processes because additives, such as initiators or catalysts, are not required [23]. The use of gamma rays as an energy source is a less harmful, less toxic, and more sustainable option for the planet because the preparation, reaction time, and chemical waste are reduced [24]. Therefore, producing high-quality and pure materials is of utmost importance to meet the worldwide demand for disposables and biomedical devices [25].

In general, grafting onto silicone films has been a good method tested on different compounds, such as one-step grafting with *N*-vinylimidazole, forming the corresponding copolymer graft poly(*N*-vinylimidazole) (PNVI) [26] or (3-mercaptopropyl)trimethoxysilane (MTS) [27], as well as in mixtures of monomers, such as hydroxyethyl methacrylate and oligo(ethylene glycol) methyl ether methacrylate silicone-g-(HEMA-co-OEGMA) [28] or with ethylene glycol dimethacrylate and glycidyl methacrylate silicone-g-(EGDMA-co-GMA) [29]. Silicone films have also been modified in a two-step grafting procedure with acrylic acid and vinyl pyrrolidone silicone-g-(AAc-g-VP) [30]. Regarding silicone catheters (SC), it is possible to find reports of grafting with 2-methacryloyloxy-benzoic acid SC-g-(2MBA) [31], acrylic acid SC-g-(AAc) [32], and *N*-vinyl caprolactam SC-g-(NVCL) [33].

One of the objectives of silicone modification is to find an adequate surface to immobilize or load biocide compounds, such as the aforementioned Ag (Figure 1B), imidazolium chains (Figure 1C), or ampicillin (Figure 1D), in order to obtain a material with improved functionality.

The proposal in this investigation is none other than the modification of a medical-grade silicone catheter with -g-PNVI through a radiation-induced graft reaction conducted at room temperature to obtain a pH-responsive biomaterial able to be functionalized via three different approaches with alkyl halides (bromoethane and iodomethane), Ag, and ampicillin to provide antimicrobial features against common nosocomial bacteria.

## 2. Results

### 2.1. Grafting NVI onto SC

**Figure 2:**
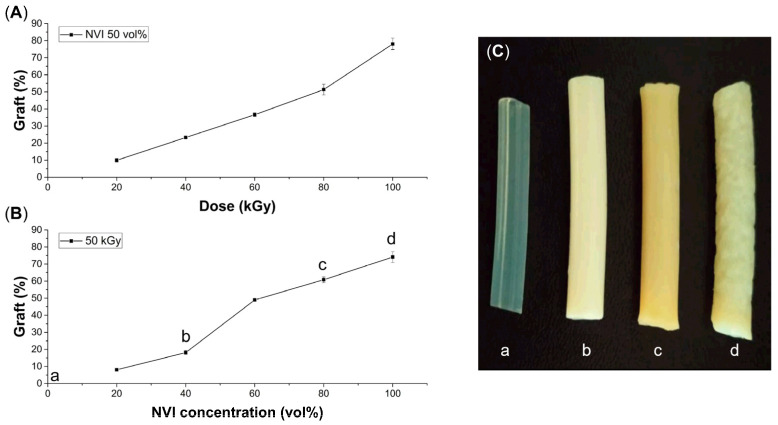
Curves of grafting of NVI onto SC depending on absorbed dose (**A**) and NVI concentration (**B**). Experiments were conducted with methanol as a solvent and irradiated with gamma rays at room temperature. (**C**) Silicone catheters with different grafting degrees of (**a**) 0%, (**b**) 18%, (**c**) 61%, and (**d**) 74%.

The modification of silicone catheters (abbreviated in this section as SC) was studied under different reaction conditions (Figure 2). In the first experimental series, the monomer concentration was 50 vol% and the absorbed dose varied from 20 to 100 kGy (Figure 2A), thus obtaining the modified silicone graft PNVI material (named SC-g-PNVI) with grafting percentages between 10 and 74%. In general, higher irradiation doses yielded higher copolymerization degrees, although this caused a size (volume) increase. As such, high irradiation doses are not recommended due to the detriments caused to catheters that make them hard and brittle. Therefore, the best grafting percentages are between 10 and 40%.

In the second series of experiments, it was decided to set the irradiation dose at 50 kGy and to vary the NVI concentration from 20 to 100 vol% in methanol as the solvent (Figure 2B). Here, it was evident that the graft percentage is favored at a higher monomer concentration, but, as with a higher dose, the SC turned off hardens, losing its original appearance and flexibility (Figure 2C). For this reason, it was determined that intermediate conditions are the best, that is, doses between 40 and 60 kGy and an NVI concentration between 40 and 60 vol%, because under these reaction conditions, acceptable grafting degrees are obtained, and the physical properties of silicone are not significantly affected.

### 2.2. Infrared (FTIR)

The FTIR spectrum of the pristine SC showed three characteristic bands of different intensities. First, at 2963 cm^−1^, there was a weak band attributed to C-H stretching of methyl; then, at 1258 cm^−1^, there was a band of intermediate intensity corresponding to the bending of the methyl group; third, there was a broad and intense band at 1005 cm^−1^, attributed to the stretching vibrations of the Si-O-Si bond. It is noted that this last band was predominant, even in the spectrum of SC-g-PNVI.

For comparative purposes, the FTIR of PNVI (isolated from crude) showed a series of characteristic bands present in the polymer. The C-H stretching vibration of the ring was observed at 3104 cm^−1^, and the aliphatic C-H stretching appeared at 2930 cm^−1^. Also, in the zone between 2500 cm^−1^ and 2000 cm^−1^ were distinguished overtones of the ring. The middle region of this spectrum also showed between 1650 cm^−1^ and 1497 cm^−1^ the C=C and C=N stretching of the ring; also, bending bands of methyl and methylene appeared at 1497 cm^−1^ and 1405 cm^−1^. Finally, secondary C-H vibrations of the C-N chain of the ring were observed at 1280 cm^−1^ and 1224 cm^−1^, and the ring bands in the fingerprint region were observed at 820 cm^−1^ and 732 cm^−1^.

**Figure 3:**
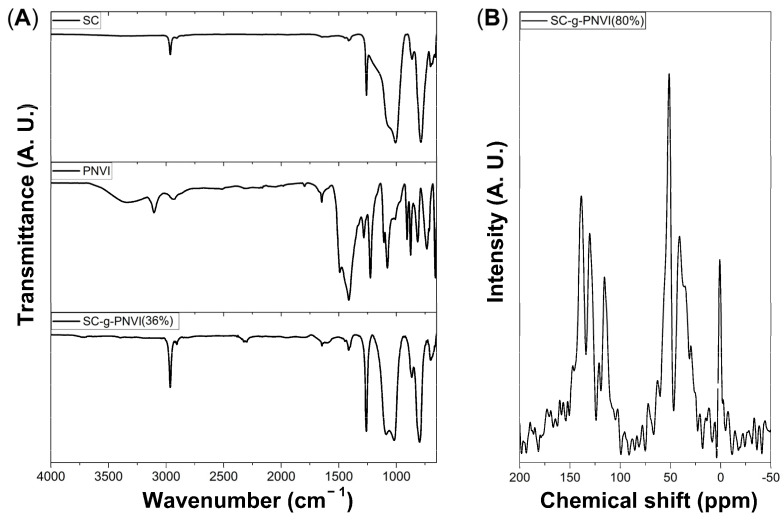
(**A**) Infrared spectra of pristine silicone catheter (SC), PNVI, and SC-g-PNVI(36%). (**B**) Solid-state spectrum ^13^C-NMR of SC-g-PNVI(80%).

In the FTIR spectrum of SC-g-PNVI, some signals expected from PNVI chains were not observed, so the resulting spectrum was comparable to silicone. Even so, the C-H stretching band was observed at 2963 cm^−1^, with more intensity than in pristine silicone; in the intermediate region, the ring overtones appeared weakly between 2500 cm^−1^ and 1600 cm^−1^, and an intense band was observed at 1258 cm^−1^ that corresponded to the contributions of the vibrational modes of methyl bending and C-N stretching (Figure 3A).

### 2.3. Solid-State ^13^C-NMR

Spectroscopic characterization of the graft copolymer was also conducted in solid-state ^13^C-NMR to confirm the structure, where all signals present in SC-g-PNVI appeared in the expected chemical shift zones. In this case, the signals of the aromatic NVI carbons appeared at 138.24, 129.99, and 115.96 ppm, while the signals of -CH-CH_2_ (backbone chain) appeared at 51.44 and 40.61 ppm, respectively. Those chemical shifts are congruent with the spectrum in liquid of PNVI [34]. Finally, the signal of silicone methyl was observed at 0.93 ppm, which is expected in aliphatic compounds.

### 2.4. Thermogravimetric Analysis (TGA)

**Figure 4:**
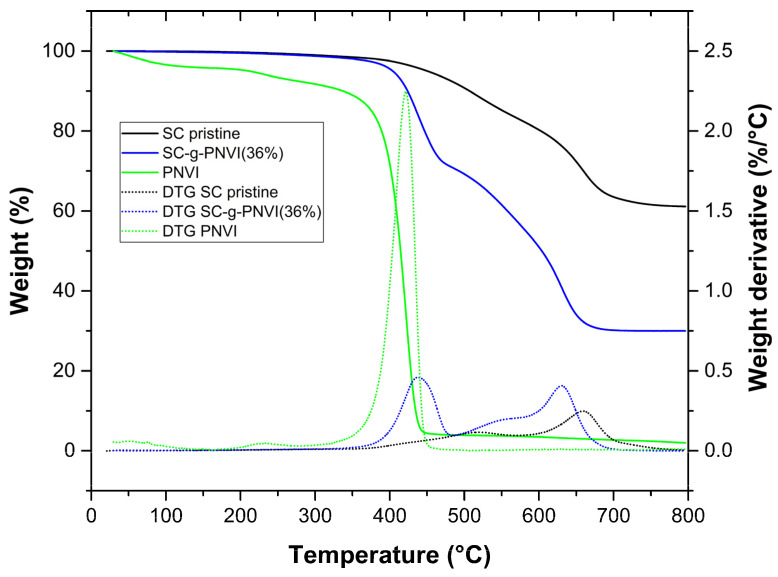
Thermograms of SC, PNVI, and SC-g-PNVI. TGA (continuous line) and DTG (dashed line) experiments were carried out at a heat rate of 10 °C/min under N_2_ atmosphere.

Figure 4 shows the thermograms of pristine SC, PNVI, and SC-g-PNVI(36%), where the decomposition temperature of the pristine SC is higher concerning the graft copolymers (Figure 4). The decomposition temperature of pristine SC was 660 °C, and the residue at 800 °C was 61.2% (mostly SiO and SiO_2_). The decomposition temperature of PNVI was 410 °C and it was rapidly consumed at higher temperatures; the char yield at 800 °C was 1.9%, a normal percentage for organic materials composed only of C, N, and H, which combustion products are volatile. Finally, for SC-g-PNVI(36%), the thermogram suggested a decomposition in two stages. The first decomposition at 437 °C corresponds to the losses of PNVI chains, and the second decomposition at 631 °C corresponds to the silicone matrix, with the residue at 800 °C of 30%.

### 2.5. Water Contact Angle

**Figure 5:**
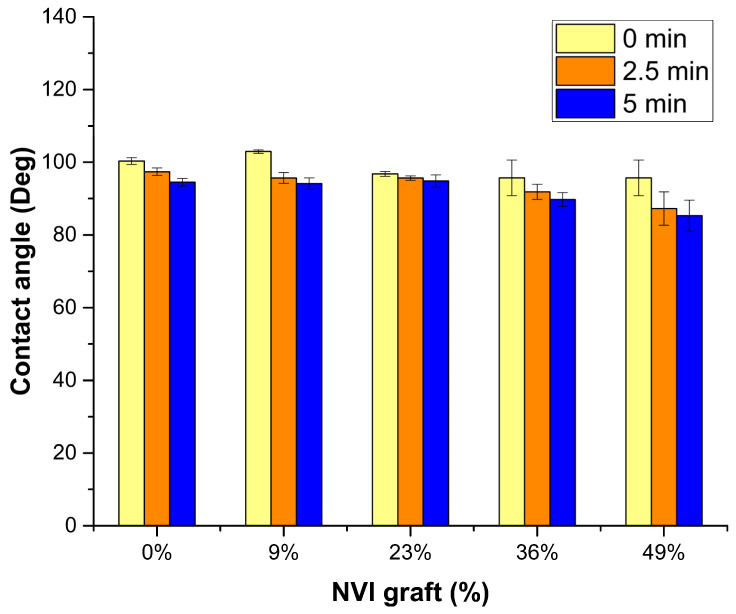
Contact angle in SC surfaces with different grafting degrees.

Bar graphs of water contact angles taken at 0, 2.5, and 5 min indicate certain similarities regardless of the grafting degree (Figure 5). The samples analyzed were pristine SC(0%) and copolymers SC-g-PNVI with various grafting degrees from 9 to 49%. The pristine silicone, as expected, presented a hydrophobic behavior with angles greater than 90°. However, the wettability in the samples grafted with 9, 23, and 36% was minimal and showed a similar angle to the pristine SC. The contact angle of the sample SC-g-PNVI(49%) was slightly hydrophilic. The high contact angles measured are attributed to the strong cohesive forces of water that, upon contact with the material surface, are unable to totally adhere.

### 2.6. Swelling and pH Response

Unlike the contact angle experiment, where the SC-g-PNVI was apparently hydrophobic, in the swelling experiments in water and phosphate buffer solution (PBS), the behavior of the grafted sample was hydrophilic as, in these experiments, the sample was completely submerged in the liquid medium such that repulsive forces were quickly overcome and the grafted material increased its affinity for water. This was different from pristine silicone, which was impermeable and did not swell in aqueous medium or PBS.

**Figure 6:**
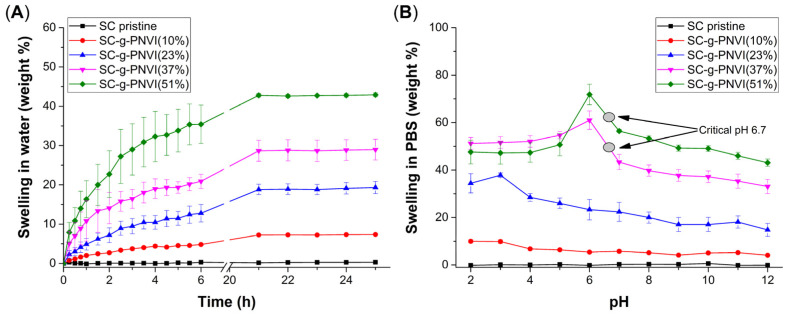
Curves of limit swelling (**A**) in water and (**B**) PBS using different grafted SC samples.

The graph of swelling on the left (Figure 6A), shows the time-dependent swelling in water at 24 h, where the maximum swelling was reached in the first 24 h. The curves of swelling on the right side show the swelling percentage in PBS medium at different pH levels (Figure 6B). Even when, in both graphs, it is apparent that the swelling degree increases with the graft percentage, particularly in PBS medium, the swelling percentages were higher at pH 6 and 7 because the critical pH was determined in this zone at pH 6.7 where swelling began to decrease, reaching the lowest swelling percentage at pH 12. This occurred because in an alkaline medium, the excess in hydroxyl ions weakly interacts with the N of PNVI chains (amines are Lewis bases) [35]. Meanwhile, in an acidic pH in the range of 2–5, the interaction among NVI groups and hydrogen ions is so strong that swollen samples break, and the real weight could not be determined. This weight loss occurred mainly in samples with the higher grafting degree of SC-g-PNVI(37% and 51%).

### 2.7. Antimicrobial Functionalization of SC-g-PNVI

**Figure 7:**
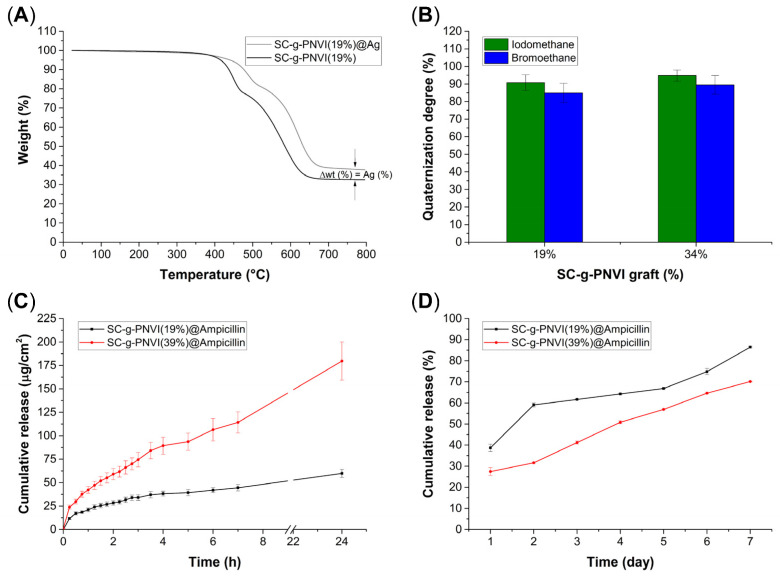
(**A**) TGA of SC-g-PNVI(19%) and SC-g-PNVI(19%)@Ag. (**B**) Quaternization degree (%) in SC-g-PNVI with iodomethane and bromoethane. (**C**) Release curves of SC-g-PNVI(19% and 39%)@Ampicillin at 24 h and (**D**) the release at 7 days.

One of the main objectives of this investigation was to test the reactivity of PNVI chains with metals, as in addition to functioning as polyelectrolytes [36], they also induce the formation of aggregates with noble metals, such as Ag [37]. In this reaction, the aqueous solution of silver nitrate darkened after a few days of contact with the catheter under sunlight, suggesting the photoreduction of Ag according to the literature [38]; in this case, the change in color only indicates that Ag is attached to the catheter surface. The quantification of immobilized Ag was carried out through TGA, where the difference between the weight percentage of the residue at 800 °C of SC-g-PNVI(19%) and SC-g-PNVI(19%)@Ag corresponds to the amount of immobilized Ag. In this case, the Ag was calculated at 5.2 weight%. Furthermore, in the thermograms, it is observed that Ag loading delayed the decomposition temperatures of the graft copolymer (Figure 7A).

The second objective of exploiting the reactivity of PNVI chains was achieved by forming imidazolium salts with iodomethane [39] and bromoethane [40]. The quaternization reaction of SC-g-PNVI(19%) and SC-g-PNVI(34%) was carried out in an aprotic medium with toluene as the solvent. The quaternization degree (%) was determined according to Equation (4), obtaining high yields as the following results suggest. SC-g-PNVI (19%) with iodomethane formed SC-g-[PNVI(19%)CH_3_]I with a quaternization degree of 91% and SC-g-[PNVI(34%)CH_3_]I with a quaternization degree of 95%, while with bromoethane it formed SC-g-[PNVI(19%)CH_2_CH_3_]Br with a quaternization degree of 85% and SC-g-[PNVI(34%)CH_2_CH_3_]Br with a quaternization degree of 90% (Figure 7B). Then, in principle, a high quaternization generates more chains with net charges, increasing in this way the electrostatic and hydrophilic interactions [41]. As such, in the presence of bacteria, the imidazolium chains generate cell membrane disruption to cause DNA damage to the microorganism [42].

Finally, the third approach consisted of the broad-spectrum drug load onto the grafted catheter; in this case, ampicillin was the model drug. Ampicillin is slightly acidic, and its carboxylic group deprotonates upon interacting with the slightly alkaline NVI in the copolymer chains (Figure 7C). The samples SC-g-PNVI(19%) and SC-g-PNVI(39%) were analyzed, and the release curves corresponded to the expected model for a polymer system in an aqueous medium through a mechanism that includes a swelling phase and Fickian diffusion [43]. Certainly, the kinetics of ampicillin release were slower than expected, as during the first 24 h for SC-g-PNVI(19%)@Ampicillin, 39% of the drug was released, while in SC-g-PNVI(39%)@Ampicillin, only 27% of the drug was released. In the long term, at 7 days, the total accumulated ampicillin release was 86% for SC-g-PNVI(19%)@Ampicillin and 70% for SC-g-PNVI(39%)@Ampicillin. Moreover, the total ampicillin loaded onto the SC-g-PNVI(19%)@Ampicillin was determined to be 155 μg/cm^2^, and for SC-g-PNVI(39%)@Ampicillin, it was 654 μg/cm^2^. Given the results, it was concluded that this antimicrobial system displays a sustained release that may be convenient for providing antimicrobial coverage during a prolonged dosage.

### 2.8. Antimicrobial Assays in Disk

**Figure 8:**
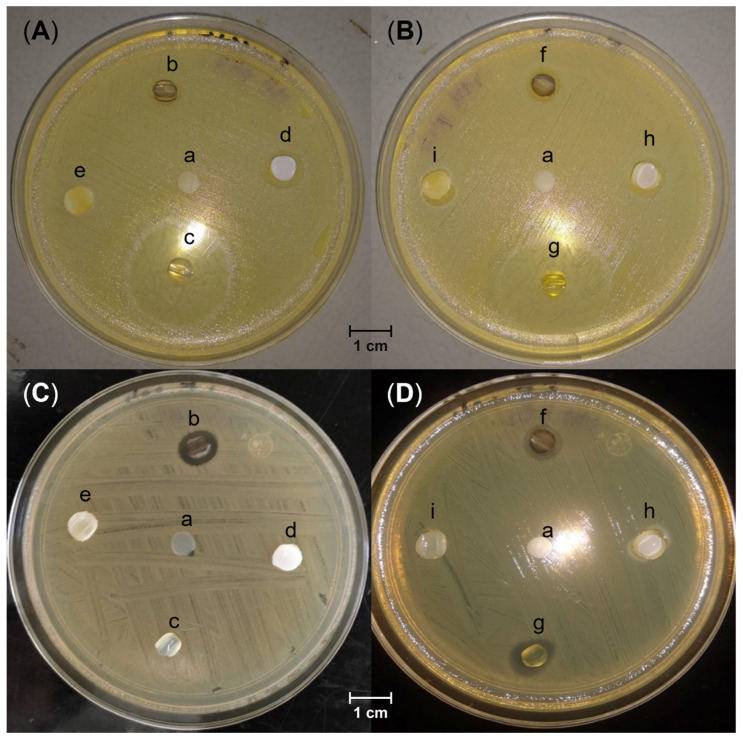
Kirby–Bauer disk diffusion susceptibility test in agar plates. (**A**,**B**) correspond to *S. aureus* strains. (**C**,**D**) correspond to *E. coli* strains. (**a**) SC; (**b**) SC-g-PNVI(19%)@Ag; (**c**) SC-g-PNVI(19%)@Ampicillin; (**d**) SC-g-[PNVI(19%)CH_3_]I; (**e**) SC-g-[PNVI(19%)CH_2_CH_3_]Br; (**f**) SC-g-PNVI(34%)@Ag; (**g**) SC-g-PNVI(39%)@Ampicillin; (**h**) SC-g-[PNVI(34%)CH_3_]I; (**i**) SC-g-[PNVI(34%)CH_2_CH_3_]Br.

The motivation for this investigation was to check the attained antimicrobial activity and compare it with the pristine SC that lacks antimicrobial protection. In this way, it was used as a control in the Kirby–Bauer disk diffusion susceptibility test (Figure 8). In summary, the functionalized samples had mixed performance in *E. coli* and *S. aureus* strains.

As a first observation, in the samples loaded with Ag, the zone of inhibition was small in both cultures of *E. coli* and *S. aureus*. Meanwhile, in the samples derivatized with bromoethane and iodomethane, SC-g-[PNVI(19%)CH_3_]I, SC-g-[PNVI(34%)CH_3_]I, SC-g-[PNVI(19%)CH_2_CH_3_]Br, and SC-g-[PNVI(34%)CH_2_CH_3_]Br, the inhibition zone was small in the disk with *S. aureus* and practically null in the disk with *E. coli*. The samples loaded with ampicillin presented great inhibitory activity against *S. aureus* but poor activity against *E. coli*.

Therefore, after evaluating the inhibitory effect of all antimicrobials, the SC-g-PNVI@Ampicillin system is the best to kill *S. aureus* selectively, and SC-g-PNVI@Ag is a moderate inhibitory system against both strains.

## 3. Discussion

Grafting: The simultaneous irradiation applied in the grafting of NVI onto SC was a method suitable for yielding high grafting percentages; however, it also caused detriments to the catheter, so choosing moderate doses and concentrations is a better option, that is, 40–60 kGy and NVI 40–50 vol%. The choice of methanol solvent and purging with Ar are not minor issues, because by changing these conditions in the reaction medium, the grafts may not be obtained.

pH response: SC-g-PNVI acquired certain hydrophilicity depending on the grafting degree, although 37% graft is adequate to determine hydrophilicity in different pH PBS media. The samples swelled more at an acidic pH and less at an alkaline pH, and the critical point was determined at pH 6.7. This value is close to a neutral pH and convenient for functionalization with Ag and ampicillin given that pH 6.7 is similar to the pH found in hypotonic and isotonic solutions used in catheters for parenteral nutrition [44].

Antimicrobial functionalization: PNVI grafted chains increased chemical activity, which for allowed the incorporation of potentially antimicrobial compounds, whether through load and release mechanisms (with ampicillin and Ag) or through derivatization with alkyl halides (iodomethane and bromoethane). After evaluating the performance of materials in vitro against bacterial strains, it was found that SC-g-PNVI@Ampicillin eradicated *S. aureus*, while SC-g-PNVI@Ag moderately inhibited the growth of both *E. coli* and *S. aureus*. Finally, the derivatization with halides yielded high quaternization rates, but the inhibition zone was barely observed in the *E. coli* culture in both bacterial cultures.

Although there were grounds in the literature to assume that any of the strategies would work to obtain an antimicrobial surface against the selected pathogens, the experimental observations differed. Because the performance of the potential antimicrobial compounds was mixed in this system, the antimicrobial disk test is suggested to verify whether bioactivity is present or not.

## 4. Materials and Methods

### 4.1. Reagents and Solvents

*N*-vinylimidazole/NVI, 99% (Sigma-Aldrich, Saint Louis, MO, USA). Medical-grade silicone catheters, 3 m, inner diameter 2.0 mm and outer diameter 5.0 mm (Goodfellow, Cambridge, UK). Ampicillin, 99% (Sigma-Aldrich, Saint Louis, MO, USA). Iodomethane, 99% (Sigma-Aldrich, Saint Louis, MO, USA). Bromoethane, 98% (Sigma-Aldrich, Saint Louis, MO, USA). Silver nitrate, 99% (Sigma-Aldrich, Saint Louis, MO, USA). Methanol, 99.5% (J.T. Bake, Mexico City, Mexico).

### 4.2. Equipment and Instruments 

Analytical balance with four decimal places (Pioneer, OHAUS; Bunkyo, Tokyo, Japan). Vacuum oven, 60 °C oven, heating baths (Yamato, ADP21; Santa Clara, CA, USA). Contact angle (Krüss, DSA 100; Matthews, NC, USA). Thermogravimetric analysis (TA Instruments, TGA Q50; New Castle, DE, USA). FTIR-ATR spectroscopy (Perkin-Elmer, Spectrum 100; ATR DiCompTM Diamond tipped glass; Norwalk, CT, USA). NMR spectroscopy (Bruker, Avance III HD 400 MHz; Billerica, MA, USA). Potentiometer (Thermo scientific, Orion Star A215; Waltham, MA, USA). Spectrophotometer UV (Analytik Jena, Specord 200 plus; Jena, Germany).

### 4.3. Synthesis of SC-g-PNVI

Monomer NVI was distilled under reduced pressure at 4 Hg mm. Silicone catheters (SC) were washed with methanol and water for 24 h and cut into small pieces (2.5 cm in length). Samples were placed in glass tubes to form the open ampoules.

In the first experimental series, 5 mL of an NVI/methanol 50% vol/vol solution was introduced into the ampoule. For the second experimental series, the monomer concentration was varied by 20, 40, 50, 60, 80, and 100% vol/vol in methanol as the solvent. Subsequently, the ampoules were deoxygenated with argon bubbling for 15 min and sealed with a flame using oxyfuel cutting equipment. The sealed ampoules were sent to the Gammabeam to be irradiated in a cobalt 60 source at a dose rate of 7.58 kGy/h. In the first experimental series, the dose was varied at 20, 40, 60, 80, and 100 kGy, while in the second experimental series, the irradiation dose was set at 50 kGy.

Once irradiated, the sealed ampoules were opened, and the grafted samples were extracted and washed in a 50% vol/vol ethanol/water bath with magnetic stirring for 24 h. If necessary, the solvent was replaced. Once cleaned, the samples were dried in an oven at 60 °C for 24 h to eliminate humidity. Finally, the weight of samples was recorded to calculate the grafting degree using the following Equation (1):

(1) $$Graft\;\left(\%\right)=100\left(\frac{w_{g}-w_{0}}{w_{0}}\right)$$

where

*w_g_* = final weight of the grafted catheter;
*w*_0_ = initial weight of the pristine catheter.

### 4.4. Characterization of SC-g-PNVI

#### 4.4.1. Experimental Details of Contact Angle

The sample was swollen and, still wet, placed between two glass plates, where it was dried at 60 °C for 72 h. The flattened surface was adequate to perform the contact angle measurements, and times were taken at 0, 2.5, and 5 min. Experiments were conducted in quintuplicate.

#### 4.4.2. Experimental Details of TGA

The samples were dried at 60 °C for 24 h before analysis, and the weight of the samples was between 15 and 20 mg. The runs were carried out under a nitrogen atmosphere from 25 to 800 °C, with a heating rate of 10 °C/min. The reported values of TGA and DTG (residue and decomposition temperature) were calculated using the native software TA Thermogravimetric Analyzer 2000.

#### 4.4.3. Experimental Details of Limit Swelling

Analysis of water absorbed was carried out using samples without grafting as well as with low and high grafting degrees. Each sample (about 50 mg) was dried at 60 °C beforehand, and the weight of the dry sample (*w*_0_) was recorded. Swelling was carried out in double-distilled water or 0.1 N PBS (pH = 2–12) as a medium and in the time determined according to each experiment. In water, the weights of the samples were recorded at different times between 0 and 24 h. In PBS medium, the time was constant and only measured at 24 h. The swelling percentage was determined through gravimetry according to the following Equation (2):

(2) $$Swelling\;\left(\%\right)=100\left(\frac{w_{s}-w_{0}\;}{w_{0}}\right)$$

where

*w_s_* = weight of the swollen sample;
*w*_0_ = weight of the dry sample.

### 4.5. Functionalization of SC-g-PNVI with Ag

SC-g-PNVI(19%) and SC-g-PNVI(34%) were cut in small circles with a 6 mm diameter, and these samples were selected for Ag loading from a source of AgNO_3_. An aqueous solution with a AgNO_3_ concentration of 1 mg/mL was prepared. A volume of 5 mL of this solution was added to a vial containing the sample. The vials were covered with parafilm and left to rest at room temperature next to a window for 72 h. Once the time was completed, the samples were extracted and washed with water to remove the non-adhered Ag. Once cleaned, the samples were dried at 60 °C and stored at room temperature until use.

Quantification of Ag immobilized onto the grafted SC was gravimetrically determined through TGA by quantifying the residue at 800 °C, according to Equation (3):

(3) $$Ag\;loaded\;\left(\%\right)=w_{R}\left(\%\right)@Ag-w_{R}\left(\%\right)$$

where

$w_{R}$(%)@Ag = residue (%) at 800 °C of the sample loaded with Ag;
$w_{R}$(%) = residue (%) at 800 °C of the sample without Ag.

### 4.6. Quaternization of SC-g-PNVI with Iodomethane and Bromoethane

Dry SC-g-PNVI(19%) and SC-g-PNVI(34%) were cut in small circles with a 6 mm diameter, and these samples were selected for derivatization with bromoethane and iodomethane. The halide solutions were prepared at a 50 vol% concentration in toluene as the solvent and with heating in a water bath at 50 °C. Once the reaction time was complete, the samples were extracted and rinsed with toluene. Drying was carried out at 60 °C inside the oven, and the samples were reserved at room temperature until use. Additional TGA was performed, and the results are in Supplementary Material S1.1.

The mathematical procedure to calculate the quaternization degree is available in Supplementary Material S2.1; below is described the found Equation (4):

(4) $$Q(\%)=100\;\left(\frac{w_{\exp halide}}{w_{theo\;halide}}\right)$$

where

*Q(%)* = quaternization degree;
*w _exp halide_* = experimental weight of the halide compound;
*w _theo halide_* = theoretical weight of the halide compound.

### 4.7. Ampicillin Loading

SC-g-PNVI(19%) and SC-g-PNVI(39%) were cut in small circles with a 6 mm diameter to perform the experiments. An aqueous solution of ampicillin with a concentration of 1 mg/mL was prepared using double-distilled water as the solvent. The solution was poured into vials with a volume of 5 mL, and then the samples were put inside the vial that was sealed with parafilm tape and placed in the sample refrigerator at 4 °C for three days. The vials were opened once the loading time was completed, and the samples were slightly rinsed with ethanol. The samples were dried in a vacuum oven at 30 °C for 24 h. The dry samples were stored at room temperature until use.

The total amount of ampicillin loaded was determined by measuring the absorbance (at 200 nm) of ampicillin released but by replacing it with known volumes (mL) of the solvent (water) until reading an absorbance of 0. In this way, the total release was forced, and the amount of ampicillin found was used to determine the cumulative release (%), shown in Figure 7D (see Supplementary Material S2.2).

### 4.8. Ampicillin Release

The previously ampicillin-loaded samples were put in vials, and 2 mL of double distilled water was added. The absorbance was measured at 200 nm until reaching 8 h. Then, it was measured again every 24 h until the release was complete. The calibration curve for the release of ampicillin obtained is shown in Equation (5):

(5) $$Absorbance=40.554\;\left[Ampicillin\right]+0.0095$$

Calculations to determine the efficiency of ampicillin release are available in Supplementary Materials Section S2.2. 

### 4.9. Kirby–Bauer Disk Diffusion Susceptibility Test

The culture medium was prepared in Hinton Müeller (Becton Dickinson Mexico, Bioxon^®^, Mexico City, Mexico). The medium was sterilized in an autoclave at 121 °C and placed in sterile Petri disks. Then, the solid agar plates were stored at 35 °C for 24 h.

*S. aureus* ATCC 25923 and *E. coli* ATCC 25922 were thawed from −70 °C to room temperature and activated with brain heart infusion broth (Becton Dickinson Mexico, Bioxon^®^, Mexico City, Mexico) and Luria broth base (Becton Dickinson Mexico, Bioxon^®^, Mexico City, Mexico), respectively. Incubation was carried out for 24 h at 35 °C. After incubation, the suspension of the microorganisms was prepared in McFarland Standard No. 0.5. The concentration of inoculation was 2.8 × 10^8^ CFU for *E. coli* and 5.4 × 10^7^ CFU for *S. aureus*. The samples were placed on the disks and incubated for 24 h at 35 °C. After the incubation period, a Vernier was used to measure the inhibition zones.

## 5. Conclusions

Gamma radiation successfully induced the grafting of NVI onto SC, and the grafted material changed its surface properties, turning it chemically active, and thus adding PNVI chains suitable for the specific purpose of achieving antimicrobial capacity. The SC-g-PNVI material is hydrophilic, pH-responsive, and behaves as a polyelectrolyte with a critical pH of 6.7. The reactivity acquired in the catheter facilitates its derivatization with alkyl halides and allows for the loading of Ag and ampicillin to endow the desired antibacterial activity. The dual function of an intravenously connected antimicrobial catheter provides added value to the engineering of medical devices as it could help to increase the chances of restoring health in catheterized patients.

## Data Availability

Contact the corresponding authors for data.

## Supplementary Materials

The following supporting information can be downloaded at: https://www.mdpi.com/article/10.3390/molecules29102225/s1, Figure S1: TGA thermograms of SC-g-PNVI and quaternized derivatives.
