# Supplementary material for: Antimicrobial Functionalization of Silicone-graft-poly(N-vinylimidazole) Catheters
Source: Molecules. 2024 May 9;29(10):2225. doi: 10.3390/molecules29102225 (PMC11123943; doi:10.3390/molecules29102225)
Supplement: Supplementary file 1 [file molecules-29-02225-s001.zip › molecules-2957884-supplementary.pdf]

molecules

MDPI

Article

Antimicrobial Functionalization of Silicone-graft-poly(*N*-vinylimidazole) Catheters

Luis Enrique Navarrete-Germán, Belén Gómez-Lázaro, Felipe López-Saucedo * and Emilio Bucio *

Departament of Radiation Chemistry and Radiochemistry, Institute of Nuclear Sciences, National Autonomous University of Mexico, Circuito Exterior s/n, Ciudad Universitaria, Mexico City C.P. 04510, Mexico; luis.german1209@gmail.com (L.E.N.-G.); belenlazaro98@gmail.com (B.G.-L.)

* Correspondence: felipelopezsaucedo@gmail.com (F.L.-S.); ebucio@nucleares.unam.mx (E.B.)

Supplementary Materials

S 1. Results

S 1.1. TGA of quaternary graft materials

S 2. Materials and methods

S 2.1. Quaternization of SC-g-PNVI with iodomethane and bromoethane

S. 2.2 Ampicillin release

S 1. Results

The information contained in this section is complementary to the TGA described in the main manuscript.

S 1.1. TGA of quaternary graft materials

The thermograms corresponding to the grafted material SC-g-PNVI(49%) and the quaternary derivatives formed with bromoethane and iodomethane are shown (Figure S1). The SC-g-PNVI(49%) only has two decomposition temperatures at 459.21 °C and 637.18 °C. For their part, the thermograms of SC-g-[PNVI(49%)CH_2CH_3]Br and SC-g-[PNVI(49%)CH_3]I are similar and present more decomposition stages. The decomposition temperatures for SC-g-[PNVI(49%)CH_2CH_3]Br were found at 343.23 °C, 377.48 °C, 448.56 °C, and 673.76 °C; while for SC-g-[PNVI(49%)CH_3]I were found at 348.53 °C, 450.52 °C, and 660.26 °C.

At the beginning of heating is observed a faster decomposition in the quaternary derivatives, but in the final part of heating, char yields (%) at 800°C are higher in the quaternized materials. Exactly the char of SC-g-[PNVI(49%)CH_2CH_3]Br was 31.3% and of SC-g-[PNVI(49%)CH_3]I was 33.3%. While the char yield of SC-g-PNVI(49%) barely reached 19.4%. The difference in the percentages of residues between the grafted material before and after quaternization can be attributed to the remanence of halogen atoms in the char heated up to 800 °C.

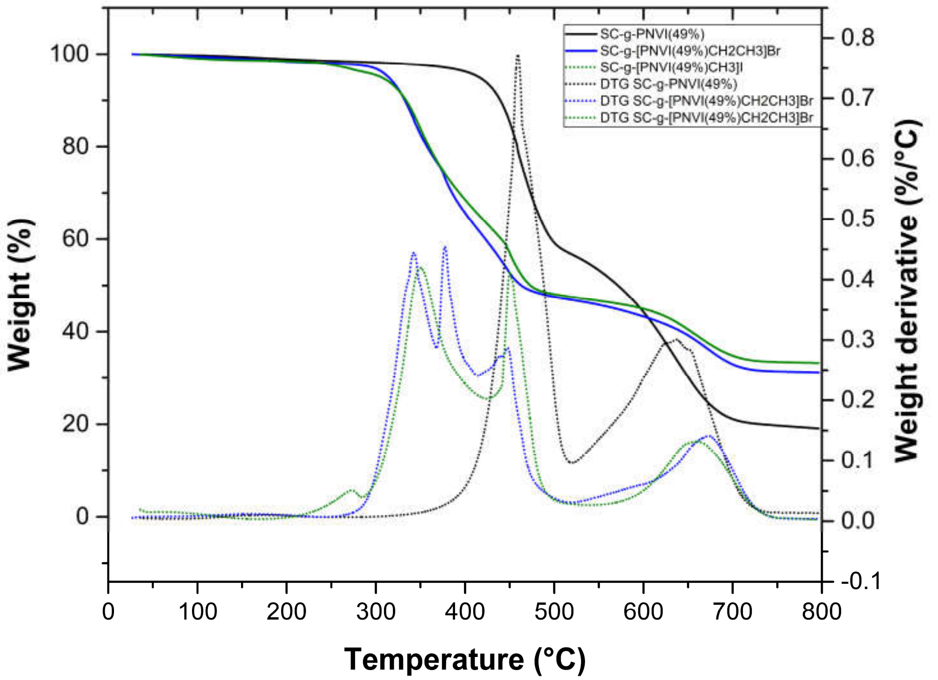

Figure S1. Thermograms of SC-g-PNVI(49%), SC-g-[PNVI(49%)CH_2CH_3]Br, and SC-g-[PNVI(49%)CH_3]I. TGA (continuous line) and DTG (dashed line) experiments were carried out at a heat rate of 10 °C/min under N_2 atmosphere.

S 2. Materials and methods

The following information supplements the data described in the main manuscript.

S 2.1. Quaternization of SC-g-PNVI with iodomethane and bromoethane

The following mathematical process was employed to calculate the quaternization degree, Q(%).

First, the weight of the pristine catheter is calculated:

$$w_0 = \frac{w_G}{\frac{G\%}{100} + 1} \quad (S1)$$

W_0= weight of pristine catheter
W_G= weight of grafted catheter
G%= graft degree

Next, the weight of PNVI grafted is calculated:

$$w_{NVI} = w_G - w_o \quad (S2)$$

W_{NVI}= weight of PNVI grafted

Subsequently, the weight of the halide is calculated assuming 100% quaternization:

$$w_{theo\ halide} = w_{NVI}\left(\frac{PM_{halide}}{PM_{NVI}}\right) \quad (S3)$$

Furthermore, using the weight of the quaternized sample, the experimental weight of the halide was calculated:

$$w_{\exp halide} = w_Q - w_G \quad (S4)$$

Finally, the quaternization percentage is calculated using the experimental and theoretical weight of the halide according to Equation S5:

$$Q(\%) = 100\left(\frac{w_{\exp halide}}{w_{theo\ halide}}\right) \quad (S5)$$

Where:

Q(%) = quaternization degree

S. 2.2 Ampicillin release

This section describes the data treatment for the quantification of ampicillin release dependent on the area. The surface of the catheter is measured with the following parameters: internal diameter, external diameter, and height. The first step is to calculate the volume of a catheter using the Equation S6:

$$V = \pi H\left(\left(\frac{D}{2}\right)^2 - \left(\frac{d}{2}\right)^2\right) \quad (S6)$$

Where:
H= catheter height
D= external diameter
d= internal diameter
Subsequently, the density is calculated using Equation S7:

$$\rho_{catheter} = \frac{w_{catheter}}{V_{catheter}} \quad (S7)$$

Hence, the volume of these can be calculated with the following Equation S8:

$$V_{samples} = \frac{w_{samples}}{\rho_{catheter}} \quad (S8)$$

And the height of the sample (h) is calculated:

$$h = \frac{V_{samples}}{\pi\left(\left(\frac{D}{2}\right)^2 - \left(\frac{d}{2}\right)^2\right)} \quad (S9)$$

Subsequently, the surface area of the sample is calculated:

$$S = 2\pi Rh + 2\pi R^2 \quad (S10)$$

$$s = 2\pi rh + 2\pi r^2 \quad (S11)$$

Where:
S= outer surface of the cylinder
s= inner surface of the cylinder

The total surface area of the cylinder is calculated with the following Equations S12-S14:

$$S_T = 2\pi hR + 2\pi hr + 2\pi R^2 - 2\pi r^2 \quad (S12)$$

$$S_T = 2\pi(Rh + rh + R^2 - r^2) \quad (S13)$$

$$S_T(mm^2) = 2\pi(h(R + r) + R^2 - r^2) \quad (S14)$$

Where:
S_T= total cylinder surface

The ampicillin concentration is calculated using the calibration curve and the absorbance of the samples:

$$[Ampicillin]\left(\frac{mg}{mL}\right) = \frac{Absorbance + 0.0095}{40.554} \quad (S15)$$

Next, the weight of ampicillin in the solution is calculated considering the volume of water added to the vials:

$$w_{ampicillin}(mg) = V_{dis}[Ampicillin] \quad (S16)$$

The next step is the calculation of the ampicillin release per surface area:

$$Release\left(\frac{mg}{mm^2}\right) = \frac{w_{ampicillin}}{S_T} \quad (S17)$$

Where:
Release = ampicillin release in surface

The units obtained in the release are converted to μg/cm²:

$$Release\left[\frac{mg}{mm^2}\right] = \left[\frac{1000\ \mu g}{1\ mg}\right]\left[\frac{100\ mm^2}{1\ cm^2}\right] = 1000 * 100 * L\ \left[\frac{\mu g}{cm^2}\right] \quad (S18)$$

Finally, Release is calculated according to Equation S19:

$$Release\ \left[\frac{\mu g}{cm^2}\right] = 1x10^5 \left(\frac{w_{ampicillin}(mg)}{S_T(mm^2)}\right) \quad (S19)$$

Author Contributions: Conceptualization, BGL and LENG; Methodology, BGL and LENG; Software, BGL, LENG, and FLS; Validation, FLS and EB; Formal analysis, FLS and LENG; Investigation, FLS and LENG; Resources, EB; Data curation, BGL, FLS, and LENG; Writing – Original draft preparation, FLS and LENG; Writing – Review & Editing, FLS and LENG; Visualization, EB; Supervision, EB; Project administration, EB; Funding acquisition, EB. All authors have read and agreed to the published version of the manuscript.

Funding: This research was funded by Dirección General de Asuntos del Personal Académico (DGAPA), Universidad Nacional Autónoma de México under grant number IN204223. FLS CVU 409872, was funded by CONAHCyT.

Institutional Review Board Statement: Not applicable.

Informed Consent Statement: Not applicable.

Data Availability Statement: Contact to correspondence authors for data.

Acknowledgments: Authors thanks to G. Cedillo from IIM-UNAM for technical assistance.

Conflicts of Interest: The authors declare no conflicts of interest.

Disclaimer/Publisher's Note: The statements, opinions and data contained in all publications are solely those of the individual author(s) and contributor(s) and not of MDPI and/or the editor(s). MDPI and/or the editor(s) disclaim responsibility for any injury to people or property resulting from any ideas, methods, instructions or products referred to in the content.
